# The potential for traditional Chinese therapy in treating sleep disorders caused by COVID-19 through the cholinergic anti-inflammatory pathway

**DOI:** 10.3389/fphar.2022.1009527

**Published:** 2022-10-10

**Authors:** Xiaoxia Xie, Nana Zhang, Jingya Fu, Zhenzhi Wang, Zirun Ye, Zhijun Liu

**Affiliations:** ^1^ Institute of Regenerative and Reconstructive Medicine, Med-X Institute, First Affiliated Hospital of Xi’an Jiaotong University, Xi’an, China; ^2^ Shaanxi University of Chinese Medicine, Xian yang, China; ^3^ National Local Joint Engineering Research Center for Precision Surgery & Regenerative Medicine, First Affiliated Hospital of Xi’an Jiaotong University, Xi’an, China; ^4^ Shaanxi Provincial Center for Regenerative Medicine and Surgical Engineering, First Affiliated Hospital of Xi’an Jiaotong University, Xi’an, China

**Keywords:** traditional Chinese therapy, cholinergic anti-inflammatory pathway, sleep disorders, coronavirus disease 2019, cytokine storms

## Abstract

Since the outbreak of Coronavirus disease (COVID-19) in 2019, it has spread rapidly across the globe. Sleep disorders caused by COVID-19 have become a major concern for COVID-19 patients and recovered patients. So far, there’s no effective therapy on this. Traditional Chinese therapy (TCT) has a great effect on sleep disorders, with rare side effects and no obvious withdrawal symptoms. The cholinergic anti-inflammatory pathway, a neuroregulatory pathway in the central nervous system that uses cholinergic neurons and neurotransmitters to suppress inflammatory responses, has been reported to be associated with sleep disorders and psychiatric symptoms. Many studies have shown that TCT activates the cholinergic anti-inflammatory pathway (CAP), inhibits inflammation, and relieves associated symptoms. Therefore, we believe that TCT may be a potential therapeutic strategy to alleviate sleep disorders induced by COVID-19 through CAP. In this review, we analyzed the relationship between cytokine storm induced by Coronavirus and sleep disorders, explained the influence of CAP on sleep disorders, discussed the TCT’s effect on CAP, and summarized the treatment effect of TCT on sleep disorders. Based on these practical researches and theoretical basis, we propose potential strategies to effectively improve the sleep disorders caused by COVID-19.

## 1 Introduction

An outbreak of pneumonia was caused by a novel coronavirus in Wuhan, Hubei Province province, China, at the end of 2019. Since then, the novel coronavirus has spread rapidly to different countries and regions and has evolved into a major international public health emergency. Clinically, the symptoms of the Coronavirus disease (COVID-19) in 2019 range from asymptomatic to mild symptoms such as fever, fatigue, and cough to severe acute respiratory distress syndrome (ARDS) (Chen et al., 2020; Kwenandar et al., 2020; Zhou et al., 2021). In addition, the COVID-19 pandemic has led to an epidemic of mental illnesses, such as insomnia, depression and anxiety, and symptoms of post-traumatic stress (Guo et al., 2020; Akıncı and Melek Başar, 2021; Huang et al., 2021). Recently, a systematic review of 10 studies using the Pittsburgh Sleep Quality Index (PSQI) questionnaire to assess sleep quality found that about a quarter of COVID-19 survivors was diagnosed with sleep disorders (Cheng et al., 2021a). Sleep disorders were the most common neuropsychiatric symptoms in patients 14–182 days after recovery from COVID-19 (Ding and Yao, 2020). The severity of COVID-19 was closely related to the intensity of the virus and the body’s inflammatory responses (Mehta et al., 2020). In severe cases, an excessive inflammatory response, known as namely, the “cytokine storm,” occurs due to the release of high levels of proinflammatory cytokines and chemokines produced by inflammatory cells. Cytokine storms can lead to multiple organ failures and even death (Channappanavar and Perlman, 2017). While many drugs are effective in relieving symptoms associated with COVID-19 (Polack et al., 2020; Doroftei et al., 2021), there have been relatively rare evidence-based assessments and interventions for mental health disorders (Lai et al., 2020).

Traditional Chinese therapy has been used in epidemic treatment for thousands of years. From smallpox and ancient plagues to avian influenza, Middle East Respiratory Syndrome (MERS), and Severe Acute Respiratory Syndrome (SARS), Chinese have extensive experience in treating infections with Traditional Chinese therapy (TCT) (Chen and Nakamura, 2004; Hsu et al., 2006; Lin et al., 2017). Traditional Chinese therapy includes acupuncture, massage, Chinese herbal medicine, ear acupuncture, moxibustion and so on. The common treatment options of TCT including acupuncture, Chinese herbal medicine and taVNS have been summarized in this paper to reveal the most promising three treatment methods. Acupuncture, Chinese herbal medicine, and transcutaneous auricular vagal nerve stimulation (taTNS) have also been explored as complementary treatments for sleep disorders, and with great effect (Lu et al., 2022; Luan et al., 2022). As a result, TCT has the potential to treat sleep disorders and psychiatric symptoms caused by COVID-19.

The cholinergic anti-inflammatory pathway (CAP) represents a neurological mechanism that suppresses inflammatory responses and was first discovered by Tracey KJ in 2000. They found that parasympathetic nervous system activity affects circulating tumor necrosis factor (TNF) concentrations and shock response to endotoxemia, a so-called “cholinergic anti-inflammatory pathway” (Qin et al., 2017). Activation of CAP is also considered a therapeutic strategy for respiratory diseases (Lv et al., 2022) and has the potential to be a promising therapeutic intervention for COVID-19 infection. The active ingredient in Chinese herbal medicine has been reported to inhibit proinflammatory cytokines and prevent cytokine storms (Dai et al., 2021; Yang et al., 2022). In addition, The World Health Organization (WHO) recommends acupuncture for 16 inflammatory diseases, and some clinical practice guidelines recommend acupuncture for multiple inflammatory diseases (Yang et al., 2016a; Wang et al., 2018a). TaVNS, derived from Chinese ear acupuncture, stimulate the auricle branch of the vagus nerve to activate CAP, which helps reduce inflammation. Several clinical and laboratory studies have also found that taVNS significantly improve and relieve inflammatory reactions (Baptista et al., 2020; Go et al., 2022a). Therefore, TCT has a high potential for treating inflammatory response symptoms caused by the novel coronavirus. In this review, we aim to analyze and summarize if TCT will be a promising strategy for the treatment of treating sleep disorders caused by COVID-19.

## 2 Methodology

The keywords “sleep disorders” was searched in PubMed and web of science from 1986 to 2022. A secondary search was conducted by screening the list of articles that met the inclusion criteria. The keywords were COVID-19, cholinergic anti-inflammatory pathway, acupuncture, taVNS and Chinese herbal medicine. The obtained articles were screened, and irrelevant title or abstract was excluded. Finally, we organized the tables, drew the figures and wrote the text to summarize the traditional Chinese therapy in treating sleep disorders caused by COVID-19 through the cholinergic anti-inflammatory pathway.

## 3 The relationship between cytokines storm and sleep disorders caused by COVID-19

Cytokines storm is essentially an immune system overreaction to infection. As the novel coronavirus enters the lungs, its S-protein specifically recognizes the host angiotensin-converting enzyme 2 receptor in alveolar epithelial type II cells. Upon binding, the host serine protease TMPRSS2 breaks down the S protein, allowing the virus to fuse with the cell membrane, and then the novel coronavirus enters the host cell (Wan et al., 2020). The host activates an immune response to clear the virus. In the early stages, virus infection causes the absorption and activation of various inflammatory cells in the lungs, releasing large amounts of cytokines and inflammatory chemokines. The TNF-α and IL-1β and other early active cytokines are rapidly secreted and peak within a few hours. Subsequently, anti-inflammatory cytokines are secreted to regulate the inflammatory response, allowing the body to eliminate harmful stimuli while maintaining cellular homeostasis. However, when the pro-inflammatory balance is disrupted, early reactive cytokines can further trigger the activation and release of a range of cytokines such as IL-2, IL-6, IL-8, IL-12, and inflammatory chemokines, leading to “cascading” effects and the uncontrolled inflammatory responses (Mehta et al., 2020). A retrospective multicenter study involving 150 COVID-19 patients suggests that virus-activated “cytokines storm syndrome” may be associated with COVID-19 mortality (Koralnik and Tyler, 2020). Meanwhile, elevated levels of serum IL-2, TNF-α, IL-7, granulocyte colony-stimulating factor, and interferon-gamma-induced protein 10 were correlated with the severity of COVID-19 (Mehta et al., 2020). Plasma levels of IL-2, IL-7, TNF-α, and other pro-inflammatory cytokines were elevated in COVID-19 patients, and levels of various inflammatory cytokines were higher in (ICU) patients than in non-ICU patients (Huang et al., 2020). Clinical studies have found that severe COVID-19 patients often experience this cytokine storm. Not only can it lead to acute lung injury, but it can also progress to multiple organs, including the central nervous system and peripheral nervous system organs (Gimeno et al., 2009; Koralnik and Tyler, 2020). Table 1 summarized the different neurological symptoms induced by COVID-19. COVID-19 patients experienced many different neurological symptoms during their illness, such as headaches, post-traumatic stress disorder (PTSD), sleep disorders, and depressive symptoms (Arnold et al., 2021; Xu et al., 2021). A previous study found that blocking the biological effects of the cytokines IL-1 and TNF can reduce the amount of non-REM sleep or NREM sleep rebound after sleep deprivation. On the other hand, increasing the supply of these cytokines promoted and inhibits NREM sleep volume and intensity. These findings suggested that both IL-1 and TNF are involved in the homeostatic regulation of sleep (Krueger and Majde, 1995; Zhang et al., 2020). In addition, anti-inflammatory cytokines IL-4, IL-10, and IL-13 were reported to reduce NREM sleep amount in rabbits (Kushikata et al., 1999; Kubota et al., 2000; Opp, 2005), while anti-inflammatory cytokines IFN-γ, IL-2, IL-6, and IL-15 promoted NREM sleep in animal models (Kubota et al., 2001a; Kubota et al., 2001b; Hogan et al., 2003). A clinical study found circulating levels of IL-1, TNF, and IL-6 peak during sleep or early morning (Lange et al., 2010; Chavan et al., 2017). Studies have shown that injecting healthy volunteers with IL-6 prolongs the NREM phase, leading to subjective fatigue and elevated CRP levels (Ranjbaran et al., 2007). In summary, high levels of inflammatory cytokines could lead to sleep disorders during COVID-19.

**TABLE 1:** Summary of the different neurological symptoms induced by the COVID-19.

Authors and publication year	Country	Sample size	Follow-up time	Experienced neurological symptoms
Jing Guo, 2020 (Guo et al., 2020)	China	2993	NA	Insomnia, depression, post-Traumatic stress symptoms, mental Health Problems
Chaolin Huang, 2020 (Huang et al., 2021)	China	1733	6 months	Sleep difficulties, anxiety and depression
Tuba Akıncı, 2020 (Akıncı and Melek Başar, 2021)	Beylikduzu	189	NA	Insomnia, anxiety and depression
Zijun Xu, 2021 (Xu et al., 2021)	China	1456	2 months	Post-traumatic stress disorder, sleep disorders, anxiety and depressive symptoms
Philip Cheng, 2021 (Cheng et al., 2021b)	America	208	1 year	Anxiety and depressive symptoms
Wenning Fu, 2020 (Fu et al., 2020)	China	1242	NA	Stress disorder, sleep disorders
Xiushi Ding, 2020 (Ding and Yao, 2020)	China	150	2 months	Anxiety, depressive symptoms
Kai Liu, 2020 (Liu et al., 2020a)	China	51	5 days	Anxiety and sleep disorders
Luisa Weiner, 2020 (Weiner et al., 2020)	China	120	6 months	Sleep disorders and depressive symptoms
Greg J Elder, 2020 (Elder et al., 2020)	England	60	8 months	Anxiety, sleep disorders, and depressive symptoms
Lígia Passos, 2020 (Passos et al., 2020)	Portugal and Brazil	550	NA	Anxiety and depressive symptoms
Lorenzo Tarsitani, 2021 (Tarsitani et al., 2021)	Italian	115	3 months	Post-traumatic stress disorder
David T Arnold, 2021 (Arnold et al., 2021)	England	110	4 weeks	Insomnia

COVID-19, coronavirus disease 2019.

## 4 The anti-inflammatory mechanism of the cholinergic anti-inflammatory pathway

**FIGURE 1:**
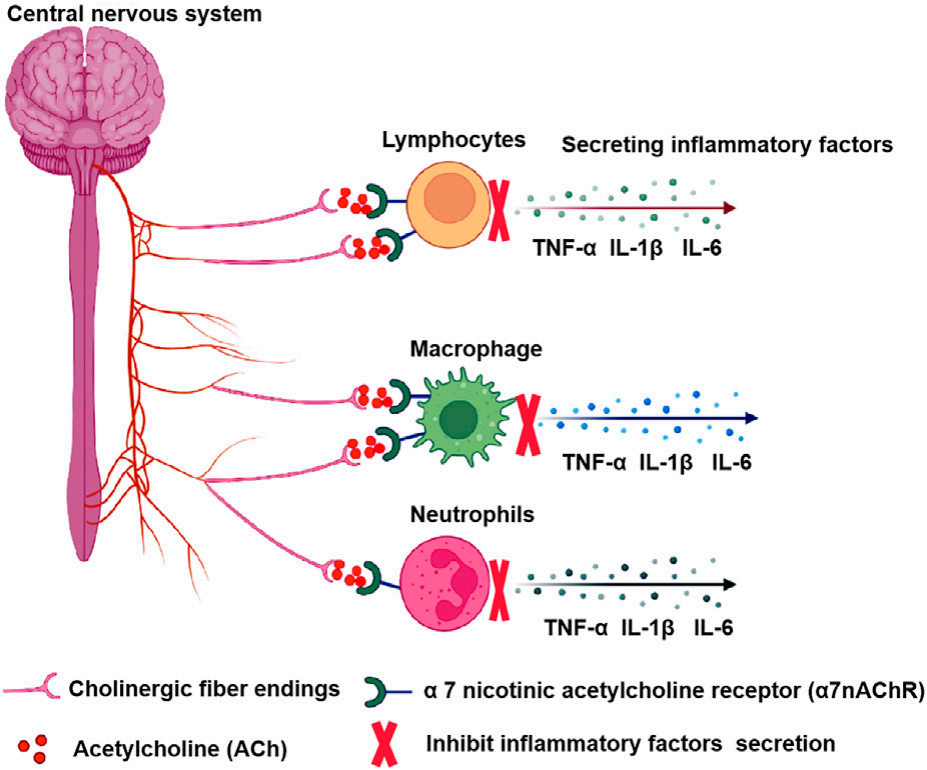
The molecular mechanisms of the CAP. The central nervous system releases ACh through efferent vagal endings and binds to α7nAChR on various inflammatory cells, inhibiting the release of pro-inflammatory factors.

CAP is a neuroregulatory pathway in the central nervous system that uses cholinergic neurons and neurotransmitters to suppress systemic inflammatory responses. It releases acetylcholine through the vented ending of efferent vagal endings and binds to α7 nicotinic acetylcholine receptor (α7nAChR) on macrophages and other immune cells, inhibiting macrophage activation and inhibiting the release of pro-inflammatory factors such as TNF-α, IL-1β, IL-6 (Pavlov et al., 2003; Pavlov and Tracey, 2015). The α7nAChR plays a key role in regulating immune responses and oxidative stress in the central and peripheral nervous systems (Ren et al., 2017), participating in processes of learning, memory consolidation, movement, and attention (Fucile et al., 2003; Park et al., 2007). α7nAChR agonist PHA-543613 reduces inflammatory damage and enhances anti-inflammatory factors and antioxidant enzymes (Xue et al., 2019). Furthermore, when the vagus nerve is electrically stimulated, the axon terminals secrete large amounts of ACh, further activating anti-inflammatory pathways in various inflammatory cells (Go et al., 2022b). Experimental results show that stimulation of the distal vagus nerve transection can prevent the elevation of liver and blood TNF caused by septic shock (Song et al., 2008). In addition, an animal model found that electrical stimulation of the vagus nerve and administration of cholinergic neurotransmitter acetylcholine inhibited levels of pro-inflammatory factor TNF-α and reduced inflammatory responses, which were exacerbated by vagotomy (Bonaz et al., 2016). Vagus nerve stimulation (VNS) also significantly reduced levels of pro-inflammatory cytokines IL-6 and IL-1β, as well as the proportion of microglia and macrophages in mice stimulated by lipopolysaccharides (Meneses et al., 2016). In summary, stimulating the vagus nerve or activating α7nAChR effectively inhibits the development of inflammation, and Figure 1 depicted the molecular mechanisms by which CAP attenuates inflammation.

Recently, activation of CAP has also been considered a strategy for the treatment of respiratory diseases (Yamada and Ichinose, 2018). The α7nAChR has been shown to activate lung resident immune cells such as alveolar macrophages, epithelial cells, and activated neutrophils, as well as slow local inflammatory responses and reduce lung injury. In the mice model of acute lung injury, VNS prevents lung injury by lung injury autonomic nervous system imbalance and activating α7nAChR through CAP (dos Santos et al., 2011; Yang et al., 2014; Liu et al., 2017a). A cohort clinical study reported the role of α7nAChR in regulating inflammatory response and oxidative stress in the chronic sleep deprivation model. Stimulation of α7nAChR contributes to adverse reactions caused by sleep deprivation. α7nAChR as a biomarker of hippocampal inflammation and oxidative stress after chronic sleep deprivation (Xue et al., 2019). Therefore, targeting CAP with VNS may be a promising treatment for lung injury and sleep disorders caused by COVID-19.

## 5 The effect of cholinergic anti-inflammatory pathway on sleep disorders

The CAP pathway is an important component of the cholinergic system that connects the nervous system to the immune system and acts as an anti-inflammatory agent through the ACh and vagus nerve (Liu et al., 2015). The cholinergic system has been reported to regulate sleep cycles (Jasper and Tessier, 1971). Studies have shown that acetylcholine plays an important role in wakefulness and breathing in people with sleep apnea (Otuyama et al., 2013). Meng et al. (2021) also found that daytime sleepiness and high blood pressure are associated with sympathetic-vagus nerve imbalance, which may be associated with decreased plasma ACh level. A clinical trial found that short sleepers responded significantly less to ACh forearm blood flow response than normal sleepers (Stockelman et al., 2021). Another study showed that ozone-induced abnormal sleep loss is associated with decreased ACh levels in the medial preoptic region rats (Alfaro-Rodríguez and González-Piña, 2005). Dexzopicclone is one of the most commonly used sleeping drugs in the clinic. It has a sedative, hypnotic effect and partially suppresses pedunculopontine tegmental (PPT) neurons by enhancing gamma-aminobutyric acid. One study reported inhibition of dextran, which reduced the release of the PPT-neuron terminals ACh in the pontine reticular formation and promoted sleep (Hambrecht-Wiedbusch et al., 2010). Cao Q Neurotransmitter test showed that saponins promoted sleep by increasing levels of acetylcholine, acetylcholine laterodorsal tegmental, and acetylcholine in PPT in mice (Cao et al., 2016). Studies have shown that cholinergic neuronal antagonists can block the activation of α7nAChR and increase the wakefulness-associated state induced by cholinergic stimulation (Zant et al., 2016). Nyctinastic herbs decoction (NHD) can prolong para-chlorophenylalanine (PCPA)-induced insomnia in mice, sleep duration, sleep quality, and depressive state was improved, and the mechanism was that the level of ACh attenuated the insomnia effect of PCPA (Yang et al., 2021). These results suggest that ACH levels play a significant role in sleep disturbance.

## 6 The effect of traditional Chinese therapy on cholinergic anti-inflammatory pathway

Some studies have found that α7nAChR plays a key role in the pathophysiology of sleep disorders and may represent a target for the treatment and control of sleep disorders (Saint-Mleux et al., 2004; Xue et al., 2019). TCT has been used in China for more than 2000 years to treat insomnia, such as acupuncture, taVNS, and Chinese herbal medicine (Sarris, 2012). However, the underlying biological mechanisms are largely unknown. Some studies have shown that they can affect CAP, suppress inflammation, and may have sleep relief (Liu et al., 2020b; Liu et al., 2021). Table 2 summarized the impact of the three most common TCT effects on CAP and their advantages and disadvantages.

**TABLE 2:** Summary of TCT’s effect on CAP and their advantages and limitations.

Types of TCT	Operational principle	Representative method	The effect on CAP[ref]	Strengths	Limitations
Acupuncture	Stimulate the specific position of the human body	Zusanli (ST36); Hegu (LI4); Shenting (GV24); Baihui (GV20)	Stimulating vagus nerve of auricle or cutaneous branch inhibits inflammatory mediators produced by macrophages (da Silva and Dorsher, 2014)	Easy; safe; less toxic; side effects	Improper operation is easy to cause infection
TaVNS	Stimulate the auricular branch of vagus nerve	Non-invasive taVNS	TaVNS can increase efferent vagus nerve excitability by stimulating auricular points in the conchal region and increase the ACh release and activate CAP (Andersson and Tracey, 2012; Pavlov and Tracey, 2012; Kaczmarczyk et al., 2017)	Convenient; easy; non-traumatic	Need professional guide
Chinese herbal medicine	Oral administration (Chinese medicine Formula and Chinese patent medicine)	Berberine; Dandelion; Chinese medicine Formula (albizzia bark, nocturnal vine, lily, and Lanzhi); Chinese patent medicine (Coptis Chinensis and cinnamon)	Inhibiting the activity of acetylcholinesterase and increasing the level of ACh and the expression of a7nAChR, thus regulating CAP and inhibiting inflammation (Li et al., 2016; Wang et al., 2018b; Wang et al., 2019; Zhao et al., 2020; Yang et al., 2021)	Efficient; multitarget; safe	Need the guidance of a professional doctor; there is a high demand for the purity of the medicine

TaVNS, transcutaneous auricular vagal stimulation; ACh, acetylcholine; CAP, cholinergic anti-inflammatory pathway.

### 6.1 Acupuncture

Acupuncture is one of the most popular complementary and alternative therapies. The efficacy of acupuncture in the treatment of inflammatory diseases has been widely reported (Li et al., 2007; Liu et al., 2022). Its anti-inflammatory effect is mainly achieved by activating the vagus nerve (Liu et al., 2013; Yu, 2022). It is performed by anatomically stimulating acupuncture points near the vagus nerve or its cutaneous branches in the ear, mastoid, and occipital regions (da Silva and Dorsher, 2014) and can be operated by manual or electrical stimulation (electroacupuncture) at different acupoints. In recent years, acupuncture has been widely recognized worldwide for its anti-inflammatory effects mediated by CAP. Acupuncture of the ear branch, which is mainly located in the stud and dorsal part of ear branch the ear, has been proven to directly affect vagus nerve activity or regulate the parasympathetic nerve (Nosadini et al., 1986; Gao et al., 2008; Imai et al., 2008; Imai et al., 2009; La Marca et al., 2010). Previous studies have found that the protective effects of electroacupuncture on the intestinal barrier are primarily associated with CAP and the reduction of inflammatory cytokines (Borovikova et al., 2000; Baek et al., 2005). In addition, acupuncture has a neuroregulatory effect on the plant nervous system and can play a role in regulating the balance of the autonomic nervous system clinically. In a mouse model of endotoxemia, Borovikova et al. found that stimulation of the vagus nerve by electroacupuncture inhibits inflammatory mediators produced by macrophages in a concentration-dependent manner (Li et al., 2015). In animal models of arthritis, electroacupuncture inhibits the production of inflammatory cytokines such as IL-1, IL-6, IL-8, and TNF through choline and reduces inflammatory pain (Cai et al., 2019; Zhou et al., 2019). Auricular acupuncture and electroacupuncture “Zusanli” (ST36) inhibited the expression of the pro-inflammatory factors TNF-α and IL-6 in rat models of endotoxemia through the cholinergic anti-inflammatory pathway (Zhao et al., 2012). In addition, electroacupuncture ST36 increased local acetylcholine transferase, promotes ACh transcription and synthesis, inhibits NF-κB expression in lung tissue, and stimulates local CAP in the lung. In another study of LPS-induced systemic infections in animals, ST36 electroacupuncture activated the vagus nerve pathway that connects the spleen, reducing the production of TNF in the spleen (Lim et al., 2016). Low-intensity electroacupuncture at ST36 acupoint in the hindlimb can effectively reduce persistent systemic inflammation (Liu et al., 2020c). Studies have also shown that electroacupuncture “Hegu” (LI4) activates muscarinic acetylcholine receptor signals in the brain through somatic afferent, and then activates the efferent vagus nerve and splenic nerve, exerting an anti-inflammatory effect, reducing TNF, IL-1β, and IL-6 levels and improving survival rate in endotoxemia model rats (Song et al., 2012). In ischemic stroke, seven consecutive days of electroacupuncture on GV20 and GV24 also increase the expression of α7nAChR in hippocampal neurons and decreased the levels of proinflammatory cytokines TNF-α and IL-1β, leading to impaired learning and memory impairment (Liu et al., 2017b). These results suggested that acupuncture’s CAP-mediated anti-inflammatory effects may improve neurological symptoms and may be an effective treatment for sleep disorders caused by COVID-19.

It is worth noting that the anti-inflammatory effect of electroacupuncture is related to acupoint selection, stimulation intensity, body condition, etc. To optimize the stimulation parameters and improve the efficacy and safety of acupuncture therapy, it is worth clinical research to investigate the stimulation intensity of electroacupuncture in driving different autonomic nerve pathways.

### 6.2 Transcutaneous auricular vagal stimulation

TaVNS comes from ear acupuncture. The ear is thought to be directly or indirectly connected to 12 meridians (six yang and six yin) (Round et al., 2013). Neuroanatomical evidence confirmed that the outer ear is the only region of the body where the vagus nerve sensory endings are located (Peuker and Filler, 2002). Recent clinical and animal experiments have shown that percutaneous auricular point vagus nerve stimulation can increase the excitability of efferent vagus nerve excitability, and increase the ACh release and CAP activation by stimulating the cochlea region. ACh binding to α7nAChR resulted in reduced secretion of inflammatory cytokines TNF, IL-1β, and IL-6 (Andersson and Tracey, 2012; Pavlov and Tracey, 2012; Kaczmarczyk et al., 2017). One study found that taVNS inhibited the expression of TNF-α, IL-1β, IL-6, and NF-kB p65 in endotoxemia rat serum through α7nAChR-mediated CAP (Jiang et al., 2018). The results implied that taVNS are a novel neurostimulation therapy with immunomodulatory and anti-inflammatory effects that may be beneficial for sleep disorders caused by inflammation caused by COVID-19.

### 6.3 Chinese herbal medicine

A large number of Chinese herbal medicine preparations for the treatment of lung diseases have an excellent effect. Activation of CAP is a theoretical basis for traditional Chinese treatment of COVID-19 infection. Berberine is an acetylcholinesterase inhibitor whose main active ingredient is derived from the Chinese herbal medicine Coptis Chinensis (Cho et al., 2006). Berberine has a neuroprotective effect by inhibiting acetylcholinesterase activity, increasing ACh levels and a7nAChR expression, thus regulating CAP, suppressing inflammation, and improving abnormal oxidative stress and cholinergic function (Li et al., 2016; Wang et al., 2019).

Jiao-Tai-Wan contains two kinds of Chinese herbal medicine: Coptis Chinensis and cinnamon. The Coptis Chinensis alkaloid is the most important component in Coptis Chinensis, possessing a variety of medicinal values. Studies have shown that berberine has antibacterial, antioxidant, cardiac, neuroprotective, and spasmodic effects (Ji and Shen, 2011; Park et al., 2012; Li et al., 2019). Cinnamon’s main active ingredient is cinnamon, which has anti-inflammatory, antioxidant, and neuroprotective effects (Yang et al., 2016b). Another study has found that Jiao-Tai-Wan activates the cholinergic pathway and improves cognitive function by reducing acetylcholinesterase activity and increasing acetylcholinesterase content (Wang et al., 2018b).

Pharmacological studies have shown that dandelions have antimicrobial, antiviral, anticancer, antioxidant, anti-inflammatory, and anti-allergic effects (He et al., 2011; Ovadje et al., 2011; Ovadje et al., 2012; Qian et al., 2014; Wang, 2014; Ma et al., 2015; Ovadje et al., 2016; Jedrejek et al., 2017; Rehman et al., 2017; Ding and Wen, 2018; Liu et al., 2018; Rahmat and Damon, 2018). Extract from Dandelion: ethyl acetate extract (EAED). Experimental results of precontraction of the tracheal ring in mice induced by high K + and ACH stimulation showed that EAED could inhibit the high concentration of Ca2+ caused by high potassium and acetylcholine. Improve airway hyperresponsiveness and reduce airway inflammation (Zhao et al., 2020). In addition, in an *in vivo* study, EAED effectively reduced ACh-induced respiratory resistance in healthy and asthmatic mice.

**FIGURE 2:**
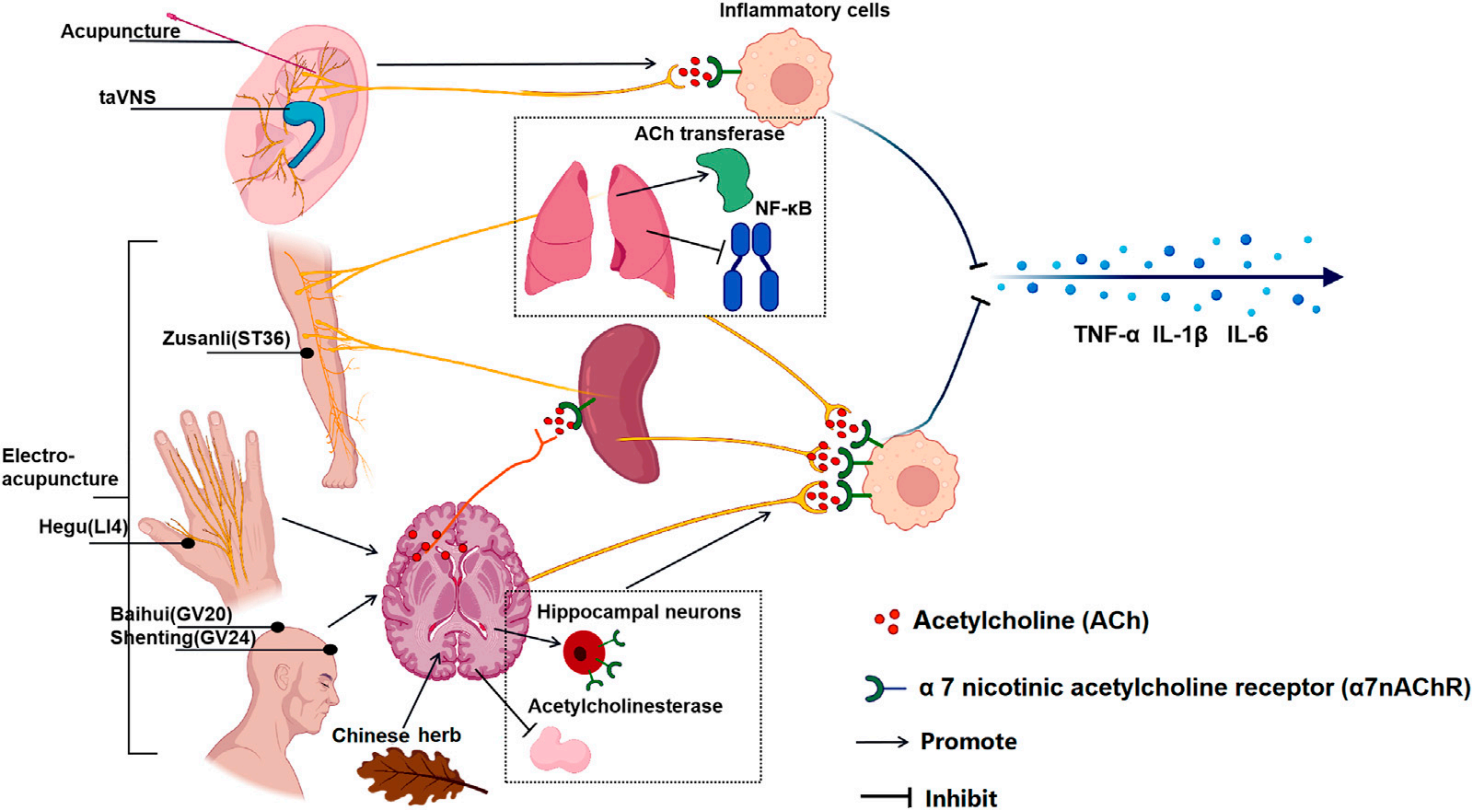
The effect of multiple TCT on CAP. Acupuncture and taVNS stimulating in ear branch can activate CAP and the reduce inflammatory cytokines; Auricular acupuncture and electroacupuncture “Zusanli” (ST36) increased the activity of local acetylcholine transferase, promoted the transcription and synthesis of ACh, inhibiting the expression of NF-κB in lung tissue and stimulating the CAP in the lung; Moreover, electroacupuncture ST36 activated the vagus nerve pathway connected to the spleen and reduced the production of TNF in the spleen; Electroacupuncture “Hegu” (LI4) activates muscarinic acetylcholine receptor signals in the brain, and then activates the efferent vagus nerve and splenic nerve to activate CAP; Electroacupuncture at “Baihui" (GV20) and “Shenting” (GV24) increase the expression of α7nAChR in hippocampal neurons; The chinese medcine Jiao-Tai-Wan reduced the activity of acetylcholinesterase (AChE) and increase the content of ACh. The ultimate effect of TCT is to activate CAP to reduce the production of inflammatory factors in effector organs to inhibit inflammation.

NHD is a traditional Chinese medicine prescription composed of albizzia bark, nocturnal vine, lily, and Lanzhi. A study of insomnia rodents induced by PCPA found that NHD has a good sedative effect in reducing exercise distance, prolonging sleep time, improving sleep quality, and improving depression, as. NHD effectively inhibits CNS excitability and relieves PCPA-induced insomnia by reducing dopamine, noradrenaline, and ACh levels (Yang et al., 2021). Figure 2 summarized the effect of multiple TCT on CAP.

## 7 The effect of traditional chinese therapy on sleep disorders

For patients with sleep disorders of different severity, the best treatment is individualized therapy tailored to each patient’s symptoms which could implement appropriate physical therapy or external treatment. TCT may be an alternative treatment for this problem. A large meta-analysis on the effects of TCT concluded that acupuncture and taVNS therapy could relieve anxiety and alleviate sleep disturbance, and even lower depression level (Wang et al., 2022). Acupuncture and Chinese herbal medicine are most commonly used in the treatment of depression-related insomnia. Studies have verified their efficacy and safety in treating insomnia (Wang et al., 2006; Song, 2007; Liu and Wan, 2010; Huo et al., 2013). Two systematic studies showed that TCT had fewer adverse reactions to insomnia than Western medicine (Yang et al., 2019; Lin et al., 2021). However, there are still some limitations in the studies related to poor methodology in Chinese herbal medicine, such as complex chemical compositions and unclear efficacy that might be the result of the comprehensive action of all drug ingredients, which to some extent restrict clear conclusions. In addition, many other TCT treatments have also improved sleep disorders (Cho et al., 2013; Bergdahl et al., 2017; Karadag et al., 2017; Li et al., 2018; Meng et al., 2018; Jiao et al., 2020; Hu et al., 2021; Jiang et al., 2021; Jiang et al., 2022a; Jiang et al., 2022b). Table 3 summarizes the effects of most TCT treatments on sleep disorders under different conditions. These studies showed that TCT possesses a great effect on sleep disorders treatment, so it should be extremely informative for sleep disorders induced by COVID-19 in the future.

**TABLE 3:** Summary for the effect of TCT on the sleep disorders.

Species and disease model	Symptoms	TCT methods	Follow-up time	Impact on sleep disorders[ref]
Human, patients In intensive care unit	Depression, anxiety, relaxation and disorders related with sleep and stress	Lavender essential oil	15 days	Alleviated patients’ stress and improved their sleep quality (Karadag et al., 2017)
Huamn, sleep disorders	Insomnia	Thermosensitive moxibustion	15 days	Improved their sleep quality (Li et al., 2018)
Mice, chronic sleep deprivation	Sleep or wakefulness disorder	Traitional medicinal herb (Dendrobium nobile Lindl extract)	2 weeks	Might be used to prevent and treat sleep or wakefulness disorder (Jiang et al., 2022a)
Human, insomnia	Insomnia	Acupuncture	12 weeks	Might adjust the emotional brain regions in adult insomnia patients, resulting in an improvement in sleep (Jiang et al., 2022b)
Human, cancer	Cancer-related fatigue, anxiety, and poor sleep quality	Traditional Chinese medicine exercise therapy (Tai Chi, Ba Duan Jin, the classics of tendon changing, Six Healing Sounds, and Wu Qin Xi)	NA	Strengthened the body and relaxed the mind, which is of significance in promoting sleep disorders (Jiang et al., 2021)
Human, insomnia	Insomnia, anxiety, depression	Chinese patent medicine (Xiao Yao San)	NA	Beneficial for improving sleep quality and relieving anxiety (Hu et al., 2021)
Human, perimenopausal insomnia	Insomnia	Auricular intradermal needling combined with erjian (HX 6,7i) bloodletting	4 weeks	Improved the sleep quality of patients with perimenopausal sleep disorders (Meng et al., 2018)
Human, insomnia	Insomnia	Auricular Acupuncture	NA	Appeared to be effective for treating insomnia (Bergdahl et al., 2017)
Human, nursing home residents with sleep disorders and psychological distress	Poor sleep quality and psychological distress	Acupressure	1 month	Improved sleep quality, reduces psychological distress (Cho et al., 2013)
Human, insomnia	Insomnia	Transcutaneous Vagus Nerve Stimulation at Auricular Concha	4 weeks	TaVNS relieved insomnia, alleviated fatigue as well as other concomitant symptoms such as depression and anxiety (Jiao et al., 2020)

In summary, high-quality sleep contributes to a strong immune system, so it is possible to resist virus invasion and kill the invaded viruses and promote the recovery of physical function. Therefore, it is worth discussing whether TCT is necessary for the treatment of the patient sleep distress. Our review firstly analyzes the reasons for the sleep disorders caused by the novel coronavirus and found that inflammation was the main reason leading to sleep distress in patients. And we reviewed the mechanisms of three common traditional Chinese in inhibiting inflammation through CAP and relieving the sleep or symptoms. We, therefore, propose that TCT may be a potential strategy to take for the treatment of sleep problems due to inflammation caused by COVID-19.

## Abbreviations

ACh, acetylcholine; ARDS, acute respiratory distress syndrome; AD: Alzheimer’s disease; α7nAChR, α 7 nicotinic acetylcholine receptor; CAP, cholinergic anti-inflammatory pathway; COVID-19, coronavirus disease 2019; CRP, C-reactive protein; EA, electroacupuncture; EAED, ethyl acetate extract of dandelion; ICU, intensive care unit; IL, interleukin; LPS, lipopolysaccharide; NHD, hypnotic decoction; NREM, non-rapid eye movements; PCPA, para-chlorophenylalanine; PPT, pedunculopontine tegmental; PSQI, pittsburgh sleep quality index; PTSD, post-traumatic stress disorder; SARS, severe acute respiratory syndrome; taVNS, transcutaneous auricular vagal nerve stimulation; TCT, traditional Chinese therapy; TMPRSS2, recombinant transmembrane protease, serine 2; TNF, tumor necrosis factor
